# High seroprevalence and associated risk factors of *Toxoplasma gondii* infection in aborted ewes from Tebessa Province, Northeastern Algeria: A One Health perspective

**DOI:** 10.14202/vetworld.2025.3367-3377

**Published:** 2025-11-06

**Authors:** Kahina Razali, Nassima Ait Issad, Faiza Mebkhout, Sofiane Boudjellaba, Djamel Khelef

**Affiliations:** 1 Institute of Veterinary Sciences, Saad Dahleb University of Blida 1, BP270, Soumaa, 09000, Blida, Algeria; 2 Laboratory of Biotechnologies Related to Animal Reproduction, Institute of Veterinary Sciences, Saad Dahleb University of Blida 1, BP270, Soumaa, 09000, Blida, Algeria; 3 HASAQ Laboratory, Higher National Veterinary School, Oued Smar, 16000, Algiers, Algeria; 4 Department of Veterinary Sciences, Higher National Veterinary School, Oued Smar, 16000, Algiers, Algeria; 5 Laboratory of Research Management of Local Animal Resources, Higher National Veterinary School, Oued-Smar, 16000, Algiers, Algeria; 6 Animal Health and Production Laboratory, Higher National Veterinary School, Oued Smar, 16000, Algiers, Algeria

**Keywords:** abortion, Algeria, One Health, risk factors, seroprevalence, sheep, *Toxoplasma gondii*, zoonosis

## Abstract

**Background and Aim:**

*Toxoplasma gondii* is an obligate intracellular protozoan responsible for reproductive losses in sheep and significant zoonotic transmission to humans. Despite its known presence in Algeria, regional epidemiological data remain inconsistent. This study aimed to determine the seroprevalence of *T. gondii* and identify associated risk factors among aborted ewes in Tebessa Province, northeastern Algeria, within a One Health framework.

**Materials and Methods:**

A cross-sectional survey was conducted from September 2019 to October 2020 across three communes (Tlidjen, Al Ater, and Negrine). Serum samples (n = 297) were collected from recently aborted Ouled Djellal ewes aged 2–5 years. Anti-*T. gondii* immunoglobulin G antibodies were detected using the Toxo-Screen DA direct agglutination test at a 1:40 dilution. Risk factors, including location, parity, gestational stage, farming system, and presence of carnivores, were analyzed by χ² tests and multivariate logistic regression using R v4.0.3.

**Results:**

The overall seroprevalence was 48.48% (144/297). Significant differences were observed across communes (Tlidjen 59.03%, Al Ater 42.72%, Negrine 30.00%; p < 0.001). Higher prevalence was recorded in sedentary (58.54%) than transhumant (26.09%) systems (odds ratio [OR] = 5.28; 95% confidence interval [CI]: 2.83–9.85; p < 0.001) and in farms with carnivores (63.31% vs. 28.91%; OR = 2.90; p < 0.001). Multiparous ewes were less likely to be seropositive than primiparous ones (OR = 0.55; p = 0.047). No significant association was found for gestation stage (OR = 1.58; p = 0.111).

**Conclusion:**

The high seroprevalence of *T. gondii* in aborted ewes indicates active environmental transmission and considerable reproductive and zoonotic risks in Tebessa. Strengthened farm biosecurity, feline population management, and public awareness of meat hygiene are urgently needed. Integrating veterinary, environmental, and public health surveillance will improve toxoplasmosis control within the One Health framework.

## INTRODUCTION

*Toxoplasma gondii* is an obligate intracellular protozoan parasite belonging to the phylum *Apicomplexa*, with felids serving as the definitive hosts and warm-blooded animals, including small ruminants and humans, acting as intermediate hosts [1, 2]. The parasite has a worldwide distribution and infects approximately one-third of the global human population [3]. It poses a considerable zoonotic threat due to its ability to form persistent tissue cysts in meat products [4]. Transmission occurs mainly through the ingestion of sporulated oocysts from contaminated environments or the consumption of undercooked meat containing cysts, while vertical transmission has also been documented in both humans and livestock [5]. The complex life cycle and remarkable environmental resilience of *T. gondii* contribute to its persistence across diverse ecosystems, reinforcing its importance in both public and veterinary health contexts [6].

In sheep, *T. gondii* infection is a major cause of reproductive losses, including abortion, stillbirth, and neonatal mortality, leading to significant economic repercussions for livestock-dependent communities [7]. Ovine toxoplasmosis is particularly devastating in regions with high seroprevalence, where repeated abortion events severely compromise flock productivity and farmer livelihoods [8]. Moreover, infected sheep serve as an important zoonotic reservoir, as the consumption of undercooked mutton remains a principal route of human infection [9]. This dual veterinary and public health impact underscores the importance of understanding the epidemiological dynamics of the parasite to mitigate its economic and health burdens.

In North Africa, and particularly in Algeria, the seroprevalence of ovine toxoplasmosis is high but varies considerably among regions, diagnostic methods, and livestock systems. Reported national estimates range from 8.28% [10] and 22.57% [10] to 35.9% [8], suggesting cumulative exposure and sustained parasite circulation. Comparable prevalence rates have been observed in neighboring countries such as Egypt (38.7%) [11] and Tunisia (19.7%) [12], reflecting the influence of ecological gradients and husbandry practices on transmission dynamics.

Despite extensive global studies on *T. gondii*, significant knowledge gaps persist regarding its epidemiology in North African small ruminant systems, particularly in Algeria. Most available studies have been limited to single provinces, relied on small sample sizes, or used heterogeneous diagnostic tests such as enzyme-linked immunosorbent assay (ELISA), modified agglutination test (MAT), indirect fluorescent antibody test (IFAT), or latex agglutination test (LAT) with varying thresholds, hindering inter-study comparability. Moreover, few investigations have specifically targeted aborted ewes, a population that provides critical insight into the reproductive and economic impact of toxoplasmosis at the flock level. Previous Algerian reports primarily estimated general flock seroprevalence without considering multivariate associations between infection risk and herd-level management variables such as farming system (sedentary vs. transhumant), feline presence, or gestational stage.

Another critical gap lies in the lack of integration of environmental and ecological determinants influencing oocyst persistence, such as humidity, temperature, and land-use patterns, which may vary substantially across Algeria’s diverse agro-ecological zones. In addition, direct quantification of oocysts in environmental matrices (soil or water) and the influence of felid population density on transmission remain poorly documented. Consequently, the absence of standardized, region-specific data constrains the development of effective biosecurity measures and One Health-based intervention programs linking animal, environmental, and human health components.

This study aimed to estimate the current seroprevalence of *T. gondii* infection in sheep from the Tebessa Province, northeastern Algeria, and to identify the major risk factors associated with its occurrence in aborted ewes. Specifically, the objectives were to:

1. Determine the prevalence of *T. gondii* antibodies in sheep using a standardized and validated serological assay (Toxo-Screen DA direct agglutination test);
2. Evaluate the association between infection and individual-level factors (parity and gestational stage) as well as herd-level determinants (geographic location, farming system, and presence of carnivores); and
3. Provide evidence-based recommendations for controlling ovine toxoplasmosis through improved farm biosecurity and integrated One Health strategies.

By addressing these aims, the present work seeks to fill the existing epidemiological gaps in Algeria, generate baseline data for risk-based surveillance, and support the formulation of targeted control policies aimed at reducing both the reproductive losses in sheep and the zoonotic burden of toxoplasmosis in rural communities.

## MATERIALS AND METHODS

### Ethical approval

All procedures complied with the Algerian National Animal Welfare Guidelines and were approved by the Algerian Directorate General for Scientific Research and Technological Development’s Institutional Animal Care Committee (Agreement Number: 45/DGLPAG/DVA.SDA.14).

This study also complies with current international principles, including the recommendations of the World Organization for Animal Health relating to the welfare, care, and use of animals in research. When animals belonging to breeders or clients were included, prior written permission was obtained from the owner after an explanation of the study objectives and sampling procedures. Owners were informed of the possibility of withdrawing their consent at any time without consequences for access to veterinary care.

### Study period and location

**Figure 1:**
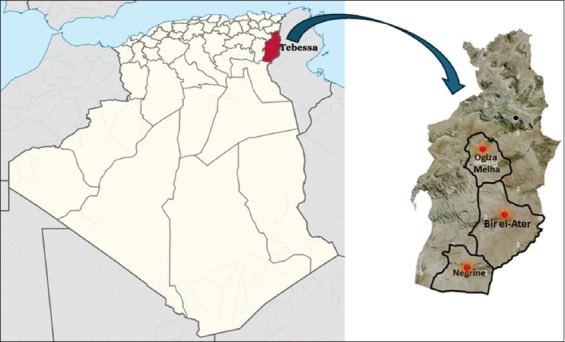
Geographical location of the study area [13].

A cross-sectional study was conducted from September 2019 to October 2020 in Tebessa Province, Algeria (35°24’15.0”N, 8°07’27.0”E) (Figure 1) [13].

The province of Tebessa is characterized by a high-altitude semi-arid steppe climate (≈880–900 m), with hot, dry summers and cold to moderately wet winters, conditions known to modulate the environmental persistence of *T. gondii* oocysts.

In Tébessa, the average annual temperature is approximately 17°C–18°C, with monthly averages ranging from approximately 7°C–8°C in winter to 29°C–30°C in summer; summer maxima frequently reach 33°C–34°C in July, while winter minima drop to approximately 2°C–4°C in January.

The mean annual rainfall is moderate to low and concentrated between autumn and spring, with typical monthly totals of approximately 20–40 mm during March–April and a strong summer drought (≈6–20 mm depending on the source), suggesting favorable seasonal windows for oocyst survival and transport during rainy events.

The mean annual relative humidity is approximately 50%–55%, ranging from approximately 33%–40% in summer to 63%–73% in late winter and spring, a gradient that may influence oocyst viability in soils and water bodies.

### Study design

This cross-sectional study used a sampling frame based on the exhaustive list of sheep farms reporting abortions to the veterinary services of the kilaya of Tébessa during the study.

Only aborted females were selected from these farms. The preferential inclusion of recently aborted ewes may artificially increase the observed serological prevalence compared with the general population of pregnant ewes or the entire flock. Future studies should adopt probability sampling, incorporating healthy and aborted ewes to limit selection bias and improve generalizability.

Individual ewe prevalence (ewe-level) was calculated as the proportion of seropositive ewes among all ewes sampled and analyzed, while flock-level prevalence (flock-level) was defined as the proportion of farms with at least one abortion rate exceeding 5% among the included farms.

No prior calculation of sample size or power analysis was performed; the sample size obtained (297 ewes, 39 flocks) resulted from pragmatic recruitment of aborted animals, constrained by the epidemic window and inter-municipal logistics, to ensure minimal representation of strategies and to detect moderate differences in seroprevalence.

This choice, centered on a descriptive objective in aborted ewes, optimizes the detection of relevant exposures but limits generalizability. The analyses integrate a random “herd” effect and structural covariates, with sensitivity analyses to contain the influence of dominant clusters.

Precision is documented by 95% confidence intervals (CIs) (overall and by strata), the minimal detectable difference is estimated a posteriori (≈15–20 points for 80% power, depending on the cluster effect), and the number of clusters/average size is reported to assess the effective precision.

### Sample collection and handling

Serum samples were collected from 297 female sheep (*Ovis aries*) of a local breed (*Ouled Djellal*), aged 2–5 years, with recent abortions (≤8 days pre-sampling) across three communes: Bir Al Ater (n = 103), Ogla Melha (n = 144), and Negrine (n = 50).

Herds were purposively selected if the abortion rate exceeded 5% [14]. The included farms typically had between 80 and 450 ewes (median ≈180), with sedentary (grazing on adjacent rangelands and daily return to pens) and transhumant (seasonal movement to wetter rangelands, shared water points) subgroups, reflecting local agropastoral practices in the Kilaya of Tébessa.

To reduce sampling bias beyond the threshold “abortions >5%”, the abortion rate on each farm had to exceed 5%, as a lower rate is considered normal and does not attract the attention of the farmer.

The health and vaccination status of the animals were documented from the breeding records and, when available, verified by veterinary certificates, including the vaccines received (antigen, type, batch, dates, schedule, route, and events/treatments of the past 30 days) for each subject.

From the selected flocks, 297 blood samples (5 mL each) were collected by jugular venipuncture from ewes kept standing, using sterile single-use needles (20–21G, 1” length) mounted on a tube holder and evacuated tubes without anticoagulant (serum separator tubes with inert gel or dry tubes with red caps), according to standard veterinary practices.

To prevent hemolysis, several precautions were taken: Avoiding multiple puncture attempts at the same site, limiting negative pressure (preferring the vacutainer system rather than vigorous aspiration with a syringe), filling tubes to their nominal volume, and keeping them upright. After collection, tubes were allowed to settle at room temperature for coagulation (≈20–30 min) without agitation and then centrifuged at 1,500 × *g* for 15 min at 4°C.

Immediately after centrifugation, sera were aliquoted into labeled cryotubes (0.5–1.0 mL per aliquot) to avoid thawing/refreezing cycles and introduced into the cold chain on refrigerated gel packs (2°C–8°C) in the shade. The time between separation and refrigeration did not exceed 30 min on site. Samples were transported daily to the provincial laboratory in approved coolers (triple packaging, absorbent, rigid container) with a temperature recorder when available. The field-to-laboratory transit time was 1–4 h, depending on the municipality.

At the laboratory, aliquots were frozen at −20°C the same day and stored without interruption of the cold chain until analysis. The median pre-test storage time was 2–6 weeks, with a maximum of 3 months, in accordance with good veterinary serum storage practices. All sera were tested in duplicate within the same run; a sample was considered positive only if both replicate wells met the 1:40 threshold. In the case of discordant replicates, the sample was reprocessed once from the original aliquot; if discordance persisted after repeat testing, the result was classified as indeterminate and excluded from inferential analyses.

### Serological testing

The choice of the direct agglutination test (DAT) and modified agglutination test (MAT) was justified by its high sensitivity, independence from species-specific reagents, operational simplicity, and strong agreement with reference methods, making it a robust and pragmatic option for multi-herd seroepidemiological surveys.

The Toxo-Screen DA agglutination test (bioMérieux, Marcy-l’Étoile, France; Cat. 73200) was used to detect anti-*T. gondii* immunoglobulin G antibodies according to the manufacturer’s recommendations [15]. Sera were separated and stored at –20°C until analysis, then diluted in phosphate-buffered saline (pH = 7.4) at 1:20 and 1:2000.

A 25 μL aliquot of each dilution was dispensed into microplate wells and supplemented with 25 μL of 0.2 M 2-mercaptoethanol (Sigma Aldrich, USA; M3148) to inactivate nonspecific IgM. A 50 μL volume of antigen suspension (formalin-fixed tachyzoites diluted 1:5 in borate buffer) was added to each well. Plates were incubated at 25°C for 5 h, and agglutination patterns were visually assessed. A diffuse sheet covering at least 50% of the bottom of the well was interpreted as a positive reaction at the 1:40 dilution threshold [16].

The 1:40 cutoff, as recommended by the manufacturer and validated in multiple studies, offers a high specificity/sensitivity balance (≈96% and ≈89%, respectively) and good comparability with ELISA and IFAT results. Systematic quality controls using positive and negative reference sera were included in each test series to verify reagent performance and incubation conditions. Repeat runs were performed in case of control non-compliance. To minimize interpretation bias, all readings were performed blindly by trained laboratory personnel.

### Risk factor data collection

Data were collected by a trained veterinarian using a standardized form completed at the time of sampling.

Individual-level variables included animal identification (tag and herd code), species and breed (Ouled Djellal ewe), estimated age (2–5 years) based on dentition and/or breeding records, reproductive status, and parity (primiparous or multiparous). The gestation stage at the time of abortion was categorized as early (days 30–90) or late (days 91–145).

Pregnancy arrests occurring before the last 2 months of gestation were rarely observed by breeders and were therefore recorded as infertility or embryonic mortality, as the embryo is reabsorbed without external expulsion.

Herd-level data included the number of animals, abortions in the past 12 months, farming system type (sedentary or transhumant), and the presence of carnivores. The presence of carnivores was defined a priori as the regular observation (≥1 cat seen on the farm within the last 7 days), with access of felids to lambing pens, feed storage areas, or water points also recorded.

Feeding and watering practices were classified as open/unprotected (ponds, uncovered troughs accessible to wildlife) or protected (covered reservoirs, raised or fenced troughs). Biosecurity measures were encoded as binary variables covering key preventive aspects: Quarantine for new animals (≥14 days), restriction of feline access to sensitive areas, isolation of sick/aborted ewes within 12 h, safe disposal of placentas/abortions, and cleaning/disinfection of lambing areas between flocks.

### Statistical analysis

Data were analyzed using R software (v4.0.3; R Foundation for Statistical Computing, Vienna, Austria). Seroprevalence was expressed with 95% CIs computed using the Wilson score method, which provides robust coverage for binomial proportions. Associations between seroprevalence and categorical risk factors were assessed using Chi-square (χ²) tests with Yates’ continuity correction for 2 × 2 tables, while Fisher’s exact test was applied when expected cell counts were <5. For binary comparisons, odds ratios (ORs) with 95% CIs were calculated from 2 × 2 contingency tables using the log-transformation method. A multivariable logistic regression model (binomial family, logit link) was developed to estimate adjusted ORs, controlling for confounders. Model adequacy was assessed through likelihood ratio tests and standard diagnostic checks. All tests were two-tailed, and statistical significance was established at α = 0.05.

## RESULTS

### Estimated seroprevalence based on different factors

In our study, the seroprevalence of *T. gondii* infection was 48.48%. Of the 297 animals sampled, 144 tested positive, indicating that nearly half of the population had been exposed to the parasite (Table 1).

**Table 1:** Seroprevalence of *Toxoplasma gondii* in aborted ewes across three communes in the Tebessa Province.

Data	Total samples	Positives	Prevalence (%)	95%CI	p-value	Pearson Chi-square	df
Total	297	144	48.48	[42.9–54.2]			
Site							
Al Ater	103	44	42.72	[33.6–52.4]	<0.001	14.6196	2
Negrine	50	15	30.00	[19.1–43.8]			
Tlidjen	144	85	59.03	[50.9–66.7]
Age							
Primiparous	203	97	47.78	[41.0–54.6]	0.723	0.1264	1
Multiparous	94	47	50.00	[40.1–59.9]
Stage							
Beginning	143	83	58.04	[49.8–65.8]	0.002	10.0852	1
End	154	61	39.61	[32.2–47.5]
Farming type							
Sedentary	205	120	58.54	[51.7–65.1]	<0.001	26.7709	1
Transhumant	92	24	26.09	[18.2–35.9]
Presence of carnivores							
Not	128	37	28.91	[21.8–37.3]	0.001	34.5225	1
Yes	169	107	63.31	[55.8–70.2]			

CI = Confidence interval, df = Degree of freedom.

#### Seroprevalence across study sites

Differences in *T. gondii* seroprevalence across the three sites were statistically significant (χ² test, p < 0.001). Tlidjen had the highest prevalence (59.03%), followed by Al Ater (42.72%) and Negrine (30.00%).

#### Parity and seroprevalence

The statistical analysis comparing *T. gondii* seroprevalence between primiparous and multiparous animals (47.78% vs. 50.00%) found no statistically significant difference (p = 0.723) (Table 1).

#### Gestational stage and seroprevalence

The difference in *T. gondii* seroprevalence between gestational stages was statistically significant (p = 0.002) (Table 1). Participants had a significantly higher prevalence (58.04%) at the beginning of gestation than at the end (39.61%).

#### Farming system and seroprevalence

The difference in *T. gondii* seroprevalence between sedentary (58.54%) and transhumant (26.09%) farming types was statistically significant (p < 0.001) (Table 1).

#### Presence of carnivores

The difference in *T. gondii* seroprevalence between areas with and without carnivores (63.31% vs. 28.91%) was statistically significant (p < 0.001) (Table 1).

#### Risk factors associated with T. gondii seropositivity in aborted ewes

Table 2 shows the results of the multivariate logistic regression analysis of risk factors associated with *T. gondii* seropositivity in sheep.

**Table 2:** Multivariate logistic regression analysis of risk factors associated with *Toxoplasma gondii* seropositivity in ewes.

Term (comparison)	Adjusted OR	95% CI	p-value	c² (df = 1)
Commune: Al Ater versus Negrine	0.72	0.40–1.28	0.266	1.24
Commune: Tlidjen versus Negrine	0.22	0.10–0.48	<0.001	10.83
Age: Multiparous versus Primiparous	0.55	0.30–0.99	0.047	3.95
Stage: Beginning versus End	1.58	0.90–2.78	0.111	2.54
Farming: Sedentary versus Transhumant	5.28	2.83–9.85	<0.001	10.83
Carnivores: Yes versus No	2.90	1.61–5.23	<0.001	10.83

OR = Odds ratio, CI = Confidence interval, df = Degree of freedom.

#### Geographic location

No significant difference was observed between Al Ater and Negrine (OR = 0.72; 95% CI: 0.40–1.28; p = 0.266). In contrast, animals from Tlidjen had a significantly lower risk than those from Negrine (OR = 0.22; 95% CI: 0.10–0.48; p < 0.001).

#### Parity

Multiparous ewes were less likely to be seropositive than primiparous ewes (OR = 0.55; 95% CI: 0.30–0.99; p = 0.047).

#### Gestational stage

The variable “Stage” was not significantly associated with infection (OR = 1.58; 95% CI: 0.90–2.78; p = 0.111).

#### Farming system

A sedentary farming system was identified as a strong risk factor compared with transhumance (OR = 5.28; 95% CI: 2.83–9.85; p < 0.001).

#### Presence of carnivores

The presence of carnivores also showed a significant effect, increasing the odds of infection by nearly threefold (OR = 2.90; 95% CI: 1.61–5.23; p < 0.001).

#### Graphical representation

**Figure 2:**
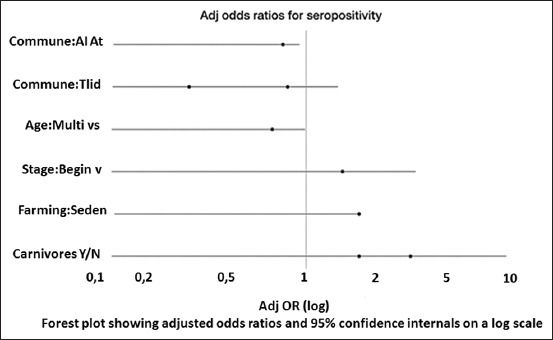
Adjusted odds ratios for *Toxoplasma gondii* seropositivity (95% confidence interval).

Figure 2 summarizes these adjusted ORs on a logarithmic scale with 95% CIs. Intervals not crossing 1 indicate statistical significance and mirror the findings, as presented in Table 2.

## DISCUSSION

### Overview and key findings

The present study identified a *T. gondii* seroprevalence of 48.48% (144/297) in sheep from Tebessa, Algeria, underscoring significant zoonotic risks for communities engaged in livestock husbandry or consuming undercooked meat. This elevated prevalence highlights the endemicity of *T. gondii* in the region, likely driven by oocyst contamination and inadequate biosecurity measures. The robust survival of oocysts in humid, temperate climates, retaining infectivity for months in soil and water, may exacerbate transmission in regions such as Tebessa, where farming practices and climatic conditions favor environmental persistence [17, 18].

### Comparison with previous studies

The observed seroprevalence exceeds earlier regional reports, including the 35.37% (133/376) reported by Benlakehal *et al*. [19] in the same province. Temporal fluctuations in environmental oocyst loads, variations in diagnostic sensitivity (e.g., indirect ELISA vs. unspecified assays), and sampling heterogeneity may explain this disparity. Notably, Benlakehal *et al*. [19] reported a 84.61% flock-level prevalence, consistent with our hypothesis of widespread environmental exposure.

Within Algeria, our findings surpass the pooled seroprevalence of 22.57% [10] and the 35.9% reported by Ouchene *et al*. [8], potentially reflecting localized ecological factors such as higher feline density or prolonged oocyst survival in humid microclimates [20].

### Global and regional context

Globally, the seroprevalence in Tebessa exceeds rates reported by Fereig *et al*. [11] in Egyptian (38.7%), by Guesmi *et al*. [12] in Tunisian (19.7%), and by Olsen *et al*. [21] in Nordic-Baltic sheep (22.7%), aligning instead with hyperendemic regions such as Saudi Arabia (68%) [22], where environmental contamination and felid activity are pronounced.

Interspecies comparisons revealed higher seroprevalence in goats (38.04–53.26%) [23, 24] than in cattle (4.4%) [25], likely due to goats’ ground-foraging behavior, which increases oocyst ingestion, versus cattle’s grazing habits and potential innate resistance [26, 27].

### Influence of animal-level factors

No association between parity and seroprevalence (p = 0.723) suggests that transmission is driven by cumulative environmental exposure rather than reproductive history. This aligns with the findings of Benlakehal *et al*. [19], emphasizing husbandry practices as primary risk factors. Conversely, the gestational stage significantly influenced seroprevalence (early: 58.04% vs. late: 39.61%; p = 0.002), possibly due to immunosuppression during early pregnancy or altered foraging behaviors increasing soil contact [26, 28].

### Influence of farming system and management practices

Sedentary farming systems exhibited markedly higher seroprevalence (58.54%) than transhumant systems (26.09%; p < 0.001), likely due to confined environments promoting oocyst accumulation in soil and water sources. This finding aligns with studies linking intensive management to elevated risk in goats [26] but contrasts with Khattak *et al*. [29], who reported higher prevalence in nomadic systems.

This discrepancy may reflect regional differences in feline density or grazing land rotation practices [20, 27]. The multivariable analysis further highlighted clear geographic heterogeneity: Sheep in Tlidjen exhibited significantly lower odds of seropositivity than those in Negrine (OR = 0.22; 95% CI: 0.10–0.48; p < 0.001), whereas no difference was observed for Al Ater relative to the reference commune (OR = 0.72; 95% CI: 0.40–1.28; p = 0.266). Beyond this spatial effect, parity was associated with reduced odds among multiparous compared with primiparous ewes (OR = 0.55; 95% CI: 0.30–0.99; p = 0.047), suggesting possible protection following prior exposures.

Regarding husbandry practices, the sedentary system emerged as a major determinant, with approximately a five-fold higher risk relative to transhumance (OR = 5.28; 95% CI: 2.83–9.85; p < 0.001), consistent with cumulative exposure on continuously grazed pastures.

The presence of carnivores on holdings was associated with a marked increase in risk, close to a three-fold elevation compared with holdings without carnivores (OR = 2.90; 95% CI: 1.61–5.23; p < 0.001), underscoring the importance of definitive hosts in environmental contamination.

In contrast, the variable “Stage Beginning versus End” was not statistically significant (OR = 1.58; 95% CI: 0.90–2.78; p = 0.111), indicating no robust difference between these categories in this dataset.

By explicitly contrasting sedentary versus transhumant husbandry, this study contributes novel evidence for North African livestock epidemiology. Sedentary systems, characterized by prolonged co-location of ewes, lambing areas, and felids, are consistent with higher cumulative environmental contamination and infection risk, whereas transhumant systems entail episodic exposures at communal pastures and watering points.

These distinct exposure ecologies have different leverage points for intervention (farm biosecurity vs. management of shared resources) and carry implications for public health risks posed by meat.

### Role of carnivores in transmission

The presence of carnivores significantly increased seroprevalence (63.31% vs. 28.91%; p < 0.001), underscoring the role of felids in oocyst dissemination. Domestic and wild felids shed millions of oocysts after infection, contaminating pastures and water [17, 30]. Disparities with goat studies, where cat presence showed weaker associations, may reflect sheep’s grazing patterns or species-specific differences in soil contact [27].

### Environmental and climatic determinants

Climate and land use critically influence transmission. Cool, moist winters, associated with prolonged oocyst survival, correlate with higher seroprevalence in cats and livestock [18]. In Tebessa, agricultural intensification and high farm density may amplify environmental contamination, as observed in French regions with similar climatic profiles [24, 31]. Furthermore, hydrological cycles can disperse oocysts into water systems, increasing livestock and human exposure [17, 32].

### Public health and One Health implications

According to the World Health Organization, approximately 22% of human *T. gondii* infections are seaborne [33]. Intermediate hosts (humans and other animals) can be infected by eating food contaminated with oocysts shed in cat feces or by ingesting tissue cysts after eating undercooked meat [34, 35].

Recent data indicate a high seroprevalence of *Toxoplasma* in small ruminants in Algeria, suggesting a significant dietary reservoir and increased risk through the consumption of contaminated meat, milk, or dairy products [10]. Some local practices include the consumption of undercooked meat; some sources even mention the traditional consumption of raw horse meat in certain regions, which increases the risk of transmission [36]. Pregnant women and immunocompromised individuals are the main vulnerable groups; high seroprevalence estimates among pregnant women and blood donors in Algeria reinforce the importance of tailored prevention messages [37]. From a One Health perspective, the results describe a transmission continuum linking animals, the environment, and humans: Small ruminant infection, facilitated by feline access to lambing, watering, and feeding areas, contributes to soil and water contamination, with direct food safety consequences for local populations.

Reducing this risk relies on coordinated actions, including on-farm biosecurity measures (protection of watering troughs, control of feline access, and safe management of abortion products), joint veterinary–environmental surveillance, and health safety communication adapted to cultural practices (thorough cooking of small ruminant meat, avoidance of raw milk).

It is proposed to deploy integrated surveillance combining periodic serology of small ruminants (prevalence and seroconversions by strata and farming system) with targeted molecular detection by quantitative polymerase chain reaction in risk matrices (abortion products, sentinel tissues, drinking water, lambing floors). Such integration would help link cumulative exposure and active contamination, identify sources, and guide prioritized on-farm biosecurity and food safety interventions [13].

### Ecological, management, and cultural interactions

The observed differences are likely explained by the interaction of ecological factors (humidity, access to water, and landscape structure), management factors (feline access to sensitive areas, lambing hygiene, placenta/abortion management, feed and water protection), and cultural factors (cat tolerance, feeding behaviors, and food preparation practices). This combination modulates both the oocyst load in the environment and the probability of animal and human exposure, justifying the need for interventions tailored to municipal and management system contexts.

### Study limitations and future research

Study limitations include geographic variability in sampling, reliance on serological assays without molecular confirmation, and insufficient data on feline reservoir dynamics. To elucidate transmission pathways, future research should integrate environmental oocyst quantification through polymerase chain reaction-based methods, spatial mapping of feline populations, and standardized diagnostic approaches. Mitigation strategies must prioritize biosecurity improvements (e.g., restricting feline access to feed/water) and public education on meat safety, particularly in high-risk pastoral communities. Although this study reports seroprevalence in aborted ewes, the generalizability of findings to the broader ovine population is limited by the absence of a formal sample size calculation and the purposive selection of animals with recent abortions.

## CONCLUSION

This study revealed a high seroprevalence of *T. gondii* infection (48.48%) among aborted ewes in Tebessa Province, Algeria, confirming the parasite’s endemic circulation in small ruminant populations and underscoring its potential impact on both animal productivity and public health. Significant differences in seroprevalence were observed across communes, farming systems, and management variables, highlighting the influence of ecological and husbandry-related determinants on transmission. Sedentary flocks, farms with feline presence, and early gestational stages exhibited notably higher infection rates, while parity and late gestation were less strongly associated. Multivariate analysis confirmed that the sedentary management system and carnivore presence were the strongest predictors of seropositivity, with five-fold and three-fold increases in odds, respectively.

The findings provide strong epidemiological evidence that *T. gondii* transmission in this region is largely sustained by environmental contamination and inadequate biosecurity practices, particularly in sedentary herds where continuous grazing and shared resources favor oocyst persistence. From a One Health perspective, these results emphasize the zoonotic potential of ovine toxoplasmosis and the importance of cross-sectoral collaboration between veterinary, environmental, and public health authorities.

Practical implications include the need for on-farm risk reduction measures, such as restricting feline access to lambing and feeding areas, safe disposal of aborted materials, disinfection of pens, and protection of water sources. Strengthening community education on the hazards of consuming undercooked meat or unpasteurized milk is equally essential to mitigate human exposure.

Study strengths lie in its focus on aborted ewes, a key sentinel population for reproductive loss, and the integration of multivariate analysis to identify risk factors under field conditions. However, limitations such as the absence of molecular confirmation and purposive sampling design restrict the generalizability of findings to the broader sheep population.

In conclusion, *T. gondii* infection remains a major but preventable threat to livestock productivity and public health in northeastern Algeria. Implementation of region-specific, evidence-based control programs, combining serological surveillance, environmental monitoring, and farmer education, will be critical to reducing infection pressure in animal populations and ensuring safer food systems under a One Health framework.

## AUTHORS’ CONTRIBUTIONS

KR and NAI: Conceived, designed, and supervised the study. KR: Performed the experiments and analyzed the data and drafted the manuscript. NAI and DK: Analyzed and interpreted the data and drafted and revised the manuscript. FM and SB: Performed the statistical analysis, interpreted the results, and contributed to the manuscript revisions. All authors have read and approved the final version of the manuscript.
